# User–fee–removal improves equity of children’s health care utilization and reduces families’ financial burden: evidence from Jamaica

**DOI:** 10.7189/jogh.07.010502

**Published:** 2017-06

**Authors:** Zhihui Li, Mingqiang Li, Günther Fink, Paul Bourne, Till Bärnighausen, Rifat Atun

**Affiliations:** 1 Department of Global Health and Population, Harvard University, Boston, Massachusetts, USA; 2 Northern Caribbean University, Mandeville, Jamaica

## Abstract

**Background:**

The impact of user–fee policies on the equity of health care utilization and households’ financial burdens has remained largely unexplored in Latin American and the Caribbean, as well as in upper–middle–income countries. This paper assesses the short– and long–term impacts of Jamaica’s user–fee–removal for children in 2007.

**Methods:**

This study utilizes 14 rounds of data from the Jamaica Survey of Living Conditions (JSLC) for the periods 1996 to 2012. JSLC is a national household survey, which collects data on health care utilization and among other purposes for planning. Interrupted time series (ITS) analysis was used to examine the immediate impact of the user–fee–removal policy on children’s health care utilization and households’ financial burdens, as well as the impact in the medium– to long–term.

**Results:**

Immediately following the implementation of user–fee–removal, the odds of seeking for health care if the children fell ill in the past 4 weeks increased by 97% (odds ratio 2.0, 95% confidence interval (CI) 1.1 to 3.5, *P* = 0.018). In the short–term (2007–2008), health care utilization increased at a faster rate among children not in poverty than children in poverty; while this gap narrowed after 2008. There was minimal difference in health care utilization across wealth groups in the medium– to long–term. The household’s financial burden (health expenditure as a share of household’s non–food expenditures) reduced by 6 percentage points (95% CI: –11 to –1, *P* = 0.020) right after the policy was implemented and kept at a low level. The difference in financial burden between children in poverty and children not in poverty shrunk rapidly after 2007 and remained small in subsequent years.

**Conclusions:**

User–fee–removal had a positive impact on promoting health care utilization among children and reducing their household health expenditures in Jamaica. The short–term and the medium– to long–term results have different indications: In the short–term, the policy deteriorated the equity of access to health care for children, while the equity status improved fast in the medium– to long–term.

User fees refer to charges related to health services at the point of use. Such fees have been used to generate revenues for health care providers, reduce health care financing burden on governments and encourage clients to use health services more judiciously [1]. Historically, both the World Bank and the International Monetary Fund (IMF) have promoted user fees [2,3]. Yet evidence points to negative effects on equitable access to health services, and arguably increased households’ health expenditures. Studies from Kenya, Tanzania, Burkina Faso, Niger, Democratic Republic of Congo, Lesotho, and Papua New Guinea have found that the introduction or increase of user fees significantly reduced health service utilization, with the poor and those in rural areas disproportionally disadvantaged because of the high financial burden [4-11]. Recognizing user fee as a barrier to access health services, the WHO passed resolutions 58.31 and 58.33 in 2005, urging member states to remove user fees in order to achieve Universal Health Coverage (UHC) [12]. UNICEF also committed to support the removal of user fees for children and pregnant women [13].

The Latin American and the Caribbean (LAC) countries have wavered between advocating or criticizing user fees over the past three decades. In the 1980s, user fees were introduced in Honduras, Jamaica, and Peru [14]. In the 2000s, Jamaica and Ecuador removed user fees in the public health sector [15]. Although a handful of studies have assessed the effects of user–fees on the quality of patient care, the work environment of health professionals, and the delivery of health services, few studies have provided concrete evidence regarding the impact of user–fee–removal policy on health care utilization and household expenditures in the LAC region [16-18].

Our study focuses on Jamaica, an upper–middle–income country in the LAC region. **Box 1** introduces Jamaica’s health system. In May 2007, the Government of Jamaica implemented a new policy that removed user fees for all children aged 0–18 years in the public sector, except for the University Hospital of the West Indies (see **Box 2**). Our study aims to evaluate the impact of user–fee–removal on children's health care utilization and household health expenditure both on average and across income groups.

Box 1 **Background information on Jamaica’s health system**

Jamaica is an upper–middle–income country with a Gross Domestic Product (GDP) per capital of US$ 8467 (constant 2011 international PPP adjusted US$) and a total population of 2.7 million in 2014. In 2014, the unemployment rate of the total labor force was 13.2% [19].

Despite moderate improvements in life expectancy, infant mortality, and under–five mortality, Jamaica has not reached the MDG4 and MDG5 targets. Before the implementation of user–fee–removal policy in 2007, Jamaica’s maternal mortality increased from 79 per 100 000 live births in 1990 to 91 per 100 000 live births in 2006. The under–5 mortality rate decreased by 36% from 30.6 per 1000 live births in 1990 to 19.5 in 2006. Infant mortality rate fell by 34% from 25.4 per 1000 live births in 1990 to 16.7 in 2006 [19].

Jamaica’s health system is financed through a mix of public and private sources. The government spends around 6% of the GDP on health. Total health expenditure per capita in 2013 was US$ 512 (constant 2011 international PPP–adjusted US$). In 2013, the government expenditure accounted for 57% of the total health expenditure and out–of–pocket payments contributed 25%, while other private sources, such as private health insurance, accounted for 18% of the total [20].

Jamaica’s public health sector is the primary provider of public health and hospital services and comprises approximately 5000 hospital beds across secondary and primary care facilities (around 1.8 hospital beds per capita). The private sector consists of approximately 200 beds (around 0.1 hospital beds per capita) and dominates ambulatory services and the provision of pharmaceuticals [20].

Box 2 **Background information on Jamaica’s user–fee–removal policy**

Historically, Jamaica’s political parties have used promise of better and lower cost health care services in campaigns to seek for votes. The removal of user fees between 2007 and 2008 in public health facilities was a practice of the campaign promise: When the People’s National Party (PNP) was in government, it has introduced the no–user–fee policy for children aged 0–18 years and considered extending no–user–fees to adults. During the General Election in Jamaica in September, 2007, the Jamaica Labour Party (JLP) made the campaign promise to remove user fees for all patients in the public health sector. After the JLP party won the 2007 General Elections, the JLP administration fulfilled its campaign promise by removing user fees in the public health sector, except at the University Hospital of the West.

In Jamaica, adjustments to user fees is nothing new, as this practice dates back to the 1960s (**Table 5**). Over the past five decades, user fees have been abolished and/or altered eight times: In 1968, Jamaica’s health authorities began revising its public health sector fee structure. User fees were removed in 1975 and reintroduced in 1984. After 23–years of user–fees in public health facilities, Jamaica abolished user fees in all public health facilities except for the University Hospital of the West Indies: on 28 May 2007, Jamaica removed user fee for children aged 0–18 years old, and on 1 April 2008, Jamaica removed user fees for adults.

**Table 5:** User fees changes in Jamaica, 1968–2008 [21]

Details	Year	In government (JLP or PNP)
Revised user fees	1968	JLP
Removal of user fees	1975	PNP
Re–introduction of user fees	1984	JLP
Adjustment of user fees (upwards)	1993	PNP
Adjustment of user fees (upwards)	1999	PNP
Adjustment of user fees (upwards)	2005	PNP
Removal of user fees (children aged 0–18 years old)	May 2007	PNP
Removal of user fees – all public patients	April 2008 – Present	JLP

JPL – Jamaica Labour Party, PNP – People’s National Party

In our study, we tested three hypotheses: First, user–fee–removal will increase health care utilization among children, because it eliminates an important barrier to access health care. Second, user–fee–removal will reduce household health expenditures in families with sick children, especially for the poor households. Third, the immediate impact of the policy may vary between children from poor families and children from better–off families and could also be different in the medium– to long–term.

Earlier studies on the impact of user–fee–removal on health care utilization and household expenditures have been mostly limited to Africa [4–10,22]. Our study is in a country of Latin America and the Caribbean, with different characteristics from Africa: Most countries in LAC belong to upper–middle– or high–income country groups and are expanding universal health coverage, with substantial social segregation and inequalities in access to health care [23,24].

Methods used in earlier studies were largely constrained by data availability, and could not identify a causal relationship between user–fee–removal policy and the changes in health care utilization, as well as households’ financial burden. We used interrupted time series (ITS) analysis to provide strong evidence for the policy’s causal effects. By comparing the changes in outcomes right before and right after the policy change, ITS analysis assumes no changes in other factors that have a potential impact on the outcomes that coincide with the policy of interest. Furthermore, ITS analysis can inform us the immediate, as well as its medium– to long–term impact of a policy.

Evaluating user–fee–removal policy for children has strong policy significance. Of all countries in the LAC region, Jamaica’s progress in reaching the Millennium Development Goals (MDGs) target for reducing child and infant mortality has been among the slowest. Between 1990 and 2006, Jamaica’s under–5 mortality rate declined by an annual rate of 2%, compared to 5% for countries in LAC and 4% in other upper–middle income countries in the world [19]. Given that child mortality is closely linked to access to health services [25,26], Jamaica’s experience can provide evidence for countries aimed at applying user–fee removal to reduce child and infant mortality. We assessed the impact of user–fee–removal policy with an equity dimension, which is a prioritized by the Sustainable Development Goals (SDGs). Our findings would shed light for the other countries on how to achieve health equity in the SDG era.

## METHODS

### Data sources

This study uses data from the Jamaica Survey of Living Conditions (JSLC) – a nationally representative household survey, which consists of six core modules: demographic characteristics, household consumption, health, education, housing, and social protection. For this paper, we use data from 1996–2012. Health module data were not collected in 2003, 2005 and 2011 surveys, and thus these waves are excluded from the study. We totally used 14 rounds of surveys in this study. Some of the earlier waves are incomplete: for example, the education level of the household head, which is an important control variable in the regression analysis, has 26.9% missing values before 2004. To solve this problem, when conducting ITS analysis, we only presented the regression results using data from 2004–2012 in the main text to ensure the key variables are with high data quality. We provided the ITS regression results using data from 1996–2012 in Tables S2, S3 and S4 in **Online Supplementary Document(Online Supplementary Document)**.

We excluded the observations interviewed within 4–weeks after the policy implementation date (28 May 2007), as it was not possible to identify whether their illness happened before or after the implementation of the policy. Moreover, subjects aged 18 years when the user fee exemption took place, were also excluded from analysis as it was difficult to ascertain whether they we over 18 or under 18 years by the time of policy change.

### Outcome variables

We have two types of outcomes: (i) health care utilization and (ii) households’ financial burden due to health care services. As with earlier studies, our measure of health care utilization is whether an individual sought care from a health professional if she/he experienced a health problem in the 4–weeks prior to the survey [27–29]. According to the JSLC, health professionals include doctors, nurses, pharmacists, midwives, healers, and other health professionals [30].

We define households’ financial burden as out–of–pocket health expenditures as a share of the household’s non–food consumption if the individual experienced a health problem in the 4–weeks prior to the survey [31]. Out–of–pocket health expenditure was defined as expenditures at public/private health centers, public/private hospitals, and costs of medicines purchased from public/private sources, which were not covered by insurance. Healthcare expenditure was considered to be catastrophic when the share of the household’s out–of–pocket health expenditure was larger than 40% of the household’s non–food consumption [22].

“People in poverty” was defined as those in the lowest wealth quintile. Utilization gap was defined as the difference in health professional visiting rates between children in poverty and children not in poverty. Gap in financial burden was defined as difference in the likelihood of encountering catastrophic health expenditure between patients from households in poverty and those not in poverty.

### Statistical analysis

We used ITS analysis to assess the impact of user–fee–removal on health care utilization, financial burden, and equity. With a clear intervention time point, ITS regressions are able to identify both immediate and medium– to long–term changes in outcomes between the pre– and post–treatment segments, assuming that no other relevant changes that might impact outcomes coincide with the policy of interest. With this feature, ITS regressions enable examination of any significant changes after the introduction of a new policy.

Our data in 2007 is from May to September, covering the exact date when the policy was implemented on 28 May 2007. We can thus directly assess the changes in health care utilization and financial burden right before and right after the implementation of the user–fee–removal policy, but also analyze medium– to longer–term impact of the policy. The ITS model used in our analysis is represented as: 

where *Y_it_* is the dependent variable for an individual observation, subscript *i* refers to the individual case and subscript *t* refers to the time, *x_it_* are the individual–level and household–level variables at time *t.* *Trend_t_* is the time variable, indicating the number of years from 2000. For example, we use 4 to represent the year 2004. *Post_t_* is the time dummy for being in the post–treatment period, estimating the immediate change of outcome when the policy occurred. The interaction term, *trend_t_* × *post_t_*, measures the change in trend in the post–intervention segment.

To further capture the policy’s impact on the equity of health care utilization and household’s financial burden, we stratify the analysis by children in poverty vs children not in poverty. Such stratification allows us to identify the effects of policy change on children from different wealth levels.

As health care utilization is a binary dependent variable, we use both ordinary least squares (OLS) and Logit regression for its analysis.

We conduct two robustness checks to ensure the results are not driven by unobservable confounders. In the first, we assume the removal of user fees in 2007 was targeted at adults over 18. If our estimates were driven by unobserved variables, such as changes in health system capacities, distance to the health facilities, opportunity cost of visiting health facilities, etc., it should also largely reflect on adults. The second robustness check assumes that the user–fee–removal policy was implemented in 28 May 2006, instead of 28 May 2007. This test could exclude the possibility that the results are driven by seasonal factors.

### Role of the funding source

There was no funding source for this study. The corresponding author obtained access to the JSLC data of year 1996–2012 via the Derek Gordon Databank. The corresponding author had full access to all the data in the study and takes responsibility for the integrity of the data and the accuracy of the data analysis as well as the decision to submit for publication.

## RESULTS

**Table 1** summarizes the individual–level and household–level characteristics in 2004, 2006, 2008, 2010 and 2012. These characteristics remain stable over years. For example, the mean age of the respondents ranges from 39.0 to 41.7 years old; male accounts for 39% to 41% of the sample, average households’ wealth quintiles range from 3.0 to 3.2.

**Table 1:** Description of key variables*

	2004	2006	2008	2010	2012
**Individual’s characteristics:**						
Age	40.7	41.7	39.1	39.0	40.5
Male	37.0%	39.0%	39.0%	40.0%	41.0%
Respondent is the head of the household	42.0%	42.0%	43.0%	41.0%	43.0%
Covered by private or public health insurance	23.0%	21.0%	23.0%	19.0%	20.0%
**Household’s characteristics:**						
Number of household members	4.2	4.2	4.2	4.0	4.0
Live in urban areas	24.0%	22.0%	31.0%	29.0%	23.0%
Live in rural areas	60.0%	59.0%	51.0%	52.0%	59.0%
Live in towns	16.0%	19.0%	19.0%	19.0%	18.0%
**Education level of the household head:**					
No education	3.0%	4.0%	2.0%	4.0%	2.0%
Primary education (Grade 1–6)	37.0%	32.0%	24.0%	27.0%	26.0%
Secondary education (Grade 7–13)	53.0%	57.0%	61.0%	58.0%	57.0%
Higher education (Grade 13+)	7.0%	7.0%	13.0%	11.0%	15.0%

*The education level of the household is obtained through the following approach: If the education level of the household head is available, we used it directly; if not available, we used the education level of the spouse of the household head instead; if still not available, we used the maximum education level of the household member instead; if still not available, we used the maximum education of the dwelling instead.

### Healthcare utilization

**Figure 1:**
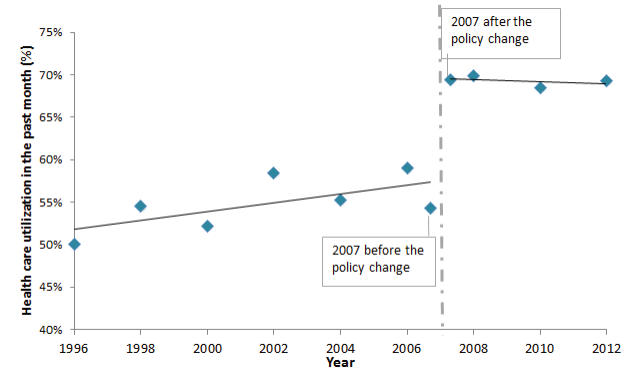
Healthcare utilization among under-18 children fell ill in the past 4 weeks. 1. To generate this figure, we split the 2007 sample into two parts–the sample interviewed before the implementation of user-fee-removal policy and the sample interviewed four weeks after it. 2. The observations numbers in the JSLC surveys vary by year (Most years have observation numbers between five thousand and eight thousand. For several years, the observation number is above fifteen thousand, such as 2008, and 2012). To increase the observation numbers involved in the generation of each data point in the figure above, we combined data from 1996 and 1997, 1998 and 1999, 2000 and 2001, 2001 and 2002, 2009 and 2010. 3. Sample weight is applied to all available years.

**Figure 1** shows children’s health care utilization over time, which largely increased from 54.2% before the implementation of user–fee–removal policy to 69.4% after the policy change in 2007. The rates remained high in the years from 2007 to 2012, ranging from 68.5% to 69.9%.

**Figure 2:**
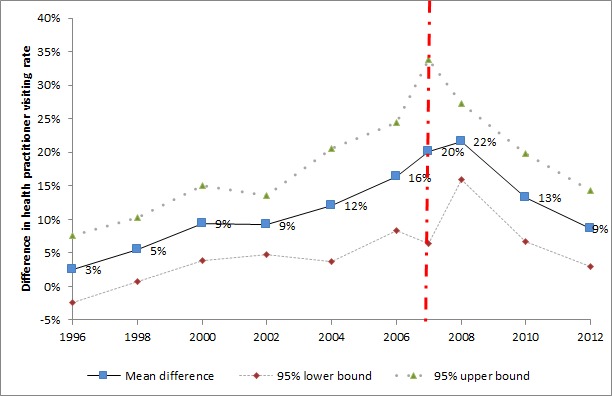
The difference in health care utilization between children in poverty and children not in poverty, among under-18 children fell ill in the past 4 weeks. 1. The observations numbers in the JSLC surveys vary by year (most years have observation numbers between five thousand and eight thousand. For several years, the observation number is above fifteen thousand, such as 2008, and 2012). To increase the observation numbers involved in the generation of each data point in the figure above, we combined data from 1996 and 1997, 1998 and 1999, 2000 and 2001, 2001 and 2002, 2009 and 2010. 2. Subjects under 18 years old in 2007 interviewed before 28 May 2007 are combined to year 2006 to prevent losing observations. 3. Sample weight is applied to all available years.

**Figure 2** presents the utilization gap – the difference in health professional visiting rates between children in poverty and children not in poverty – with 95% confidence intervals (CI). Before the policy change in 2007, the utilization gap gradually rose from 2.6% to 16.4% between 1996 and 2006. In the short–term (2007–2008), the utilization gap further increased and reached 21.7% in 2008. However, this trend reversed in the medium– to long–term (after 2008) as the children in poverty increased their utilization at a higher rate than the children not in poverty. The utilization gap shrank by nearly two–thirds between 2008 and 2012, and reached 8.7% in 2012.

**Table 2** presents the ITS regression results among individuals aged less than 18–years old (columns 1–3) and children aged less than 5 years old (columns 4–6). Column 1 and 4 shows the results for all children of that age group. Columns 2 and 3, as well as columns 5 and 6, stratify the children by wealth and show the regression results for children in poverty and children not in poverty respectively.

**Table 2:** ITS regression on the impact of user–fee–removal policy on health care utilization among children less than 18–years and children aged less than 5 years (Logit regression, presented in odds ratio and 95% CI)*

	Under 18 years old			Under 5 years old		
	**(1)**	**(2)**	**(3)**	**(4)**	**(5)**	**(6)**
	**Overall (OR, 95% CI)**	**In poverty (OR, 95% CI)**	**Not in poverty (OR, 95% CI)**	**Overall (OR, 95% CI)**	**In poverty (OR, 95% CI)**	**Not in poverty (OR, 95% CI)**
Trend	1.09 (1.00–1.18)†	0.9 (0.73–1.12)	1.13 (1.01–1.27)†	1.16 (0.95–1.42)	1 (0.67–1.48)	1.16 (0.96–1.42)
Post	1.97 (1.12–3.46)†	1.47 (0.23–9.45)	1.82 (1.10–3.00)†	4.54 (0.98–21.16)‡	7.17 (0.44–117.88)	2.93 (0.70–12.20)
Post×trend	0.95 (0.89–1.02)	1.08 (0.83–1.40)	0.94 (0.87–1.02)	0.85 (0.67–1.06)	0.87 (0.54–1.42)	0.88 (0.73–1.05)
Age	0.95 (0.94–0.97)§	0.98 (0.90–1.06)	0.95 (0.93–0.97)‡	0.84 (0.79–0.90)‡	0.84 (0.73–0.95)‡	0.84 (0.79–0.89)‡
Male	0.95 (0.82–1.09)	0.9 (0.52–1.55)	0.98 (0.79–1.21)	0.95 (0.74–1.23)	0.78 (0.39–1.57)	1.04 (0.78–1.39)
Enrolled in private health insurance	1.70 (1.18–2.44)§	1.45 (0.49–4.31)	1.82 (1.13–2.93)†	1.11 (0.76–1.62)	0.11 (0.02–0.56)‡	1.48 (0.81–2.69)
Enrolled in public health insurance	1.91 (0.83–4.43)	4.01 (1.71–9.44)‡	1.67 (0.63–4.46)	2.53 (0.98–6.52)‡	13.16 (3.77–45.93)‡	1.65 (0.50–5.45)
Wealth (the poorest wealth quintile is the reference group):						
Poorer	1.18 (0.90–1.55)			1.32 (0.75–2.33)		
Middle	1.55 (1.27–1.90)§			1.75 (1.10–2.78)†		
Richer	1.90 (1.34–2.69)§			2.11 (1.16–3.81)†		
Richest	1.72 (1.17–2.55)§			2.33 (1.10–4.96)†		
Household size, members only	0.97 (0.93–1.01)‡	0.95 (0.85–1.07)	0.95 (0.89–1.01)‡	0.97 (0.90–1.05)	0.96 (0.79–1.17)	0.95 (0.87–1.03)
Place of residence (“rural” is the reference group):						
Urban	1.18 (0.77–1.82)	1.01 (0.44–2.32)	1.29 (0.79–2.11)	1.11 (0.70–1.76)	1.4 (0.49–3.99)	1.14 (0.70–1.83)
Town	1.06 (0.65–1.71)	0.92 (0.50–1.70)	1.13 (0.72–1.78)	1.04 (0.58–1.87)	0.62 (0.18–2.12)	1.29 (0.75–2.21)
Education level of the head of the household (“no education” is the reference group)‖:						
Primary education (Grade 1–6)	0.61 (0.48–0.76)§	0.25 (0.14–0.45)§	1 (0.65–1.54)	0.55§ (0.40–0.76)	0.17 (0.07–0.42)§	1.19 (0.75–1.88)
Secondary education (Grade 7–13)	0.66 (0.45–0.97)§	0.34 (0.22–0.53)§	0.95 (0.51–1.75)	0.76 (0.51–1.12)	0.36 (0.19–0.70)§	1.12 (0.63–1.98)
Higher education (Grade 13+)	0.56 (0.36–0.89)†	0.44 (0.19–1.02)‡	0.76 (0.41–1.40)	0.67 (0.40–1.10)	0.42 (0.20–0.87)†	0.97 (0.51–1.84)
Cons.	1 (0.56–1.77)	5.78 (1.18–28.39)†	0.92 (0.48–1.74)	0.83 (0.19–3.57)	5.94 (0.42–84.18)	0.95 (0.23–3.87)
N	1931	441	1488	959	237	722

OR – odds ratio, CI – confidence interval

*The design of JSLC is a two–stage stratified random sampling design, with the first stage a selection of Primary Sampling Units (PSUs), and the second stage a selection of dwellings. Standard errors are clustered at sampling region level, which is one level above the PSUs. Two PSUs were grouped into one sampling region. The robust standard errors are reported in parentheses.

†Significance at the 1% level.

‡Significance at the 5% level.

§Significance at the 10% level.

‖The education level of the household is obtained through the following approach: If the education level of the household head is available, we use it directly; if not available, we use the education level of the spouse of the household head instead; if still not available, we use the maximum education level of the household member instead.

The implementation of user–fee–removal policy in 2007 immediately and significantly increased the odds of health care utilization by 97% (OR = 2.0, 95% CI 1.1 to 3.5, *P* = 0.018) among all children aged less than 18 years. The stratified regressions show that children not in poverty significantly increased the odds of seeking for health care when fell ill by 82% (OR = 1.8, 95% CI 1.1 to 3.0, *P* = 0.005) following the policy change. There is no significant change to the health care utilization among children in poverty. A joint F–test in columns 2 and 3 rejected the null hypothesis that two models are the same (F = 135, *P* < 0.001).

Columns 4–6 are the results for children aged under 5 years. As shown in column 4, the odds of health care utilization increased by 354% (OR = 4.5, 95% CI 1.0 to 21.2, *P* = 0.054) after the policy change among all children aged less than 5 years. The stratified results in column 5 and 6 show that the magnitudes of “post” are large for both wealth groups, yet the effects are insignificant. A joint F–test on the results shown in columns 5 and 6 rejected the null hypothesis that two models are the same (F = 586, *P* < 0.001).

The results from the OLS regressions are very consistent with the OLS regression results (see Table S1 in **Online Supplementary Document(Online Supplementary Document)**). These results are also consistent with the regression results with data from 1996 to 2012 (see Table S2 and S3 in **Online Supplementary Document(Online Supplementary Document)).**

### Healthcare expenditures

**Figure 3:**
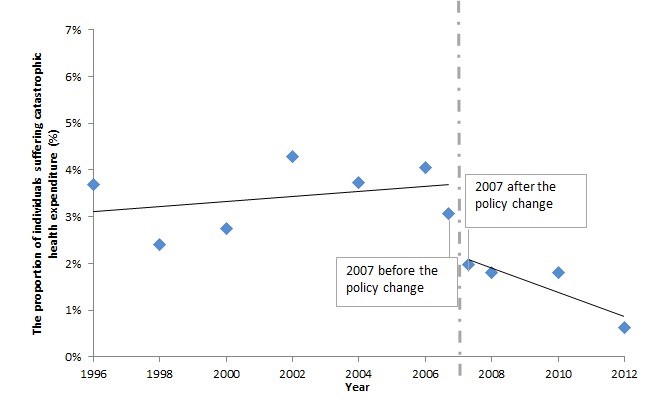
The proportion of households with under-18 children suffered catastrophic health expenditure in the 4 weeks preceding the survey if the children fell ill in the past 4 weeks. 1. To generate this figure, we split the 2007 sample into two parts–the sample interviewed before the implementation of user-fee-removal policy and the sample interviewed four weeks after it. 2. The observations numbers in the JSLC surveys vary by year (most years have observation numbers between five thousand and eight thousand. For several years, the observation number is above fifteen thousand, such as 2008, and 2012). To increase the observation numbers involved in the generation of each data point in the figure above, we combined data from 1996 and 1997, 1998 and 1999, 2000 and 2001, 2001 and 2002, 2009 and 2010. 3. Sample weight is applied to all available years.

**Figure 3** presents the percentage of households with sick children suffering from catastrophic health expenditure. We observe that the proportion of households with sick children suffering from catastrophic health expenditure immediately reduced from 3.1% to 2.0% after the policy change in 2007. The percentage of households with sick children suffering from catastrophic health expenditure continued to decline between 2007 and 2012. In 2012, only 0.6% of households with children aged less than 18–years encountered catastrophic health expenditure.

**Figure 4:**
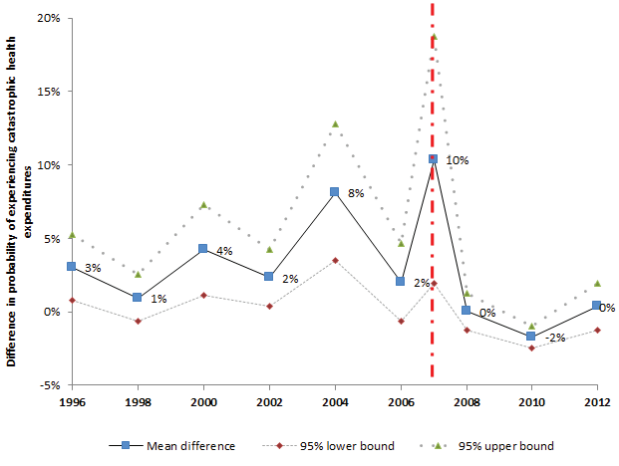
Difference in probability of experiencing catastrophic health expenditures between households in poverty and households not in poverty with sick children. 1. The observations numbers in the JSLC surveys vary by year (Most years have observation numbers between five thousand and eight thousand. For several years, the observation number is above fifteen thousand, such as 2008, and 2012). To increase the observation numbers involved in the generation of each data point in the figure above, we combined data from 1996 and 1997, 1998 and 1999, 2000 and 2001, 2001 and 2002, 2009 and 2010. 2. Subjects under 18 years old in 2007 interviewed before 28 May 2007 are combined to year 2006 to prevent losing observations. 3. Sample weight is applied to all available years.

**Figure 4** presents the financial burden gap, which is the difference between households in poverty and households not in poverty with sick children to encounter catastrophic health expenditures. The financial burden gap reduced rapidly in the short–term (2007–2008) and remained low in the medium– to long–term. In 2008, households in poverty, for the first time in the year analyzed, became no more likely to encounter catastrophic health expenditures than the households not in poverty. Such a phenomenon is also observed in years 2010 and 2012.

**Table 3** shows the ITS regression results on the household’s financial burden. The first three columns cover children aged less than18–years and the last three columns refer to children aged less than 5 years. The results show that the user–fee–removal policy significantly reduced financial burden by 6.2 percentage points (95% CI –11 to –1, *P* = 0.02) among children under 18–years. The stratified regressions show that the policy change reduced the financial burden significantly by 12.1 percentage points (95% CI –22 to –2, *P* = 0.02) among children in poverty and 5 percentage points (95% CI –12 to 2, *P* = 0.133) among children not in poverty.

**Table 3:** ITS regressions on impact of user–fee–removal policy on out–of–pocket health care expenditure as a share of household’s non–food consumption*

	Aged less than 18 years			Aged less than 5 years		
	**1)**	**2)**	**3)**	**4)**	**5)**	**6)**
	Overall	In poverty	Not in poverty	Overall	In poverty	Not in poverty
Trend	–0.003 (0.004)	–0.007 (0.007)	–0.002 (0.006)	–0.001 (0.007)	0.001 (0.015)	0 (0.008)
Level change after user–fee–removal policy (post)	–0.062 (0.023)†	–0.121 (0.045)†	–0.051 (0.031)	–0.071 (0.036)‡	–0.091 (0.085)	–0.057 (0.040)
Trend change after user–fee–removal policy:						
(Post×trend)	0.006 (0.004)	0.013 (0.011)	0.004 (0.006)	0.005 (0.007)	0.008 (0.015)	0.002 (0.008)
Age	–0.001 (<0.001)§	0.000 (0.001)	–0.001 (<0.001)§	–0.003 (0.001)†	–0.004 (0.002)‡	–0.003 (0.001)†
Male	–0.004 (0.003)	–0.006 (0.009)	–0.004 (0.003)	–0.006 (0.007)	–0.001 (0.016)	–0.008 (0.008)
Head of the household	0.015 (0.066)	–0.073 (0.034)‡	0.100 (0.008)§			
Enrolled in private health insurance	–0.011 (0.006)‡	0.032 (0.026)	–0.021 (0.006)§	–0.017 (0.009)‡	–0.025 (0.010)†	–0.022 (0.009)†
Enrolled in public health insurance	–0.011 (0.008)	–0.015 (0.008)†	–0.014 (0.011)	–0.007 (0.011)	–0.005 (0.014)	–0.015 (0.014)
Wealth (the poorest wealth quintile is the reference group)†:						
Poorer	0.003 (0.004)			0.004 (0.005)		
Middle	0.008 (0.004)‡			0.01 (0.007)		
Richer	0.004			0.003		
	(0.009)			(0.012)		
Richest	–0.008			–0.009		
	(0.006)			(0.006)		
Household size, members only	–0.006 (0.001)§	–0.002 (0.002)	–0.007 (0.001)§	–0.006§ (0.001)	–0.002 (0.002)	–0.008 (0.002)§
Place of residence (“rural” is the reference group):							
Urban	–0.005 (0.004)	–0.015 (0.012)	–0.003 (0.004)	–0.007 (0.005)	–0.016 (0.020)	–0.006 (0.004)
Town	–0.007 (0.002)§	–0.004 (0.004)	–0.008 (0.003)§	–0.004 (0.003)	0.005 (0.007)	–0.008 (0.003)†
Education level of the head of the household (“no education” is the reference group)‖:						
Primary education (Grade 1–6)	–0.005 (0.006)	–0.023 (0.014)	0.000 (0.008)	–0.01 (0.013)	–0.045 (0.020)†	0.006 (0.009)
Secondary education (Grade 7–13)	–0.010 (0.004)†	–0.021 (0.011)‡	–0.007 (0.007)	–0.009 (0.005)	–0.024 (0.015)	–0.005 (0.009)
Higher education (Grade 13+)	–0.008 (0.004)‡	0.006 (0.012)	–0.013 (0.008)	–0.008 (0.006)	–0.011 (0.015)	–0.009 (0.009)
cons	0.128 (0.022)§	0.133† (0.056)	0.132 (0.034)§	0.132§ (0.040)	0.099 (0.085)	0.135 (0.049)†
r^2^¶	0.076	0.062	0.1	0.094	0.076	0.132
N	1921	439	1482	951	234	717

*The design of JSLC is a two–stage stratified random sampling design, with the first stage a selection of Primary Sampling Units (PSUs), and the second stage a selection of dwellings. Standard errors are clustered at sampling region level, which is one level above the PSUs. Two PSUs were grouped into one sampling region. The robust standard errors are reported in parentheses. We excluded the top 1% of individuals with the highest health care cost (outliers).

†Represents significance at the 5% level.

‡Represents significance at the 10% level.

§Represents significance at the 1% level.

‖The education level of the household is obtained through the following approach: If the education level of the household head is available, we use it directly; if not available, we use the education level of the spouse of the household head instead; if still not available, we use the maximum education level of the household member instead; if still not available, we use the maximum education of the dwelling instead.

¶r^2^ represents the adjusted R square.

Columns 4–6 are the results for children aged less than 5 years. As shown in column 4, the share of out–of–pocket health care expenditure in household’s non–food consumption reduced by 7.1 percentage points (95% CI –15 to 1, *P* = 0.075) after the policy change among all children aged less than 5 years. The stratified results in columns 5 and 6 show negative, yet insignificant magnitudes of “post”. Joint F tests on the results shown in columns 2 and 3, as well as columns 4 and 5, rejected the null hypothesis that the models are the same (F = 194, *P* < 0.001; F = 167, *P* < 0.001). These results are consistent with the regression results using data from year 1996 to 2012 (see Table S4 in **Online Supplementary Document(Online Supplementary Document)**).

### Robustness check

To make sure that unobservable confounders do not drive our results, we conducted two robustness checks: First, we assume the removal of user fees in 2007 was targeted at adults aged more than 18–years. Table S5 in **Online Supplementary Document(Online Supplementary Document)** presents the regression results of the test. As expected, we can see that the coefficients on “post” and “post×trend” are neither with large magnitudes nor statistically significant, indicating that the policy change in 2007 did not have any notable impact on the adults aged more than 18–years in terms of health care utilization and financial burden.

Second, we assume the user–fee–removal policy was implemented on 28 May 2006, instead of 28 May 2007. Tables S6, S7 and S8 in **Online Supplementary Document(Online Supplementary Document)** presents the results of using the alternative starting date. None of the coefficients on “post” and “post*trend” are with large magnitudes or statistically significant, suggesting the robustness of our findings.

## DISCUSSION

**Figure 1** shows that the implementation of user–fee–removal policy in Jamaica led to increased children’s health care utilization immediately after the introduction of the policy and the utilization remained high in the medium– to long–term. This finding is consistent with earlier studies elsewhere that elimination of user fees could effectively promote utilization because it removes financial barrier to access health care [1–10].

The OLS regressions in Table S1 in **Online Supplementary Document(Online Supplementary Document)** suggest that health care utilization increased by 15.8 percentage points among children aged less than 18–years and 32.5 percentage points among children aged less than 5 years. In fact, a large proportion of children’s deaths are preventable and curable, for example, the 2005 MICS survey showed that 35% of Jamaican girls and 60% of the Jamaican boys with suspected pneumonia were not treated with potentially life–saving antibiotics [32]. Better health care access is an essential factor to save these lives [33].

**Figure 2**, combined with the ITS results in **Table 2**, implies that the short–term and the medium– to long–term results appear to have different equity impact: In the short–term (2007–2008), the utilization gap enlarged due to the faster increase in health care utilization among children not in poverty compared to children in poverty. One potential explanation for this observation is that wealthier households are better at receiving information about new policies and tend to be quicker in changing their behavior in the short–term. While in the medium– to long–term (after 2008), **Figure 2** further indicates that the utilization gap decreased rapidly as the utilization by children in poverty increased at a faster pace than non–poor between 2008 and 2012. This finding suggests that while conducting equity analysis, one should pay special attention to the study period, because various lengths of studies could produce different results.

We find that the user–fee–removal policy significantly reduced the share of out–of–pocket health care expenditure in households’ non–food consumption by 6.2 percentage points among children aged less than 18–years and 7.1 percentage points among children aged less than 5 years. The children in poverty appear to benefit more than the children not in poverty, which indicates that the policy had a larger effect to relieve the financial burden of the poor. Our results are consistent with earlier studies undertaken elsewhere, demonstrating that user–fee exemptions reduce part of financial barriers for patients, and help improve access to health services [34–37].

The study has four potential limitations. First, we cannot conclusively determine whether the increase in health care utilization was due to the release of unmet demand or moral hazard. When health services become free or inexpensive, people may tend to overuse them, leading to wastage of health resources. Whether this happened in the case of Jamaica and the extent to which it changed people’s behavior is unclear. Second, due to the limited sample size, we are not able to conduct an analysis on the health care utilization among infants and can neither draw any conclusion on the link between the policy change and health outcomes. If more comprehensive data with larger sample size were available, more detailed analysis would be possible. Third, health care expenditure data are not collected yearly, but with a 4–week recall period. We adjusted the yearly non–food consumption to reflect the 4–week period. This method may generate biased estimates if the non–food consumption is not evenly divided over months or if children are more or less likely to be sick in the months the surveys were conducted. Fourth, although two sets of robustness checks were conducted, this study is still observational and could not completely rule out the possibility of confounders.

Notwithstanding these limitations, however, our results are in line with earlier studies undertaken elsewhere and strongly confirm the effectiveness of user–fee–removal policies in improving the equal access to health care for children by promoting the equitable utilization of health services and reducing the financial burden which households may confront [1–10]. An important implication of our results is that removing user fees is feasible and should be considered as part of a potential strategy to achieve UHC. Our results also suggest that the effects of policies may change over time. Hence, policymakers should take both short–term and the long–term effects into consideration when designing user–fee policies.

## References

[1] Lagarde M, Palmer N (2011). The impact of user fees on access to health services in low-and middle-income countries.. Cochrane Database Syst Rev.

[2] Yates R (2009). Universal health care and the removal of user fees.. Lancet.

[3] John EU (2013). The impacts of user fees on health services in sub-Saharan African countries: a criticical analysis of the evidence.. Am J Public Health Res.

[4] Nabyonga J, Desmet M, Karamagi H, Kadama PY, Omaswa FG, Walker O (2005). Abolition of cost-sharing is pro-poor: evidence from Uganda.. Health Policy Plan.

[5] Pariyo GW, Ekirapa-Kiracho E, Okui O, Rahman MH, Peterson S, Bishai DM (2009). Changes in utilization of health services among poor and rural residents in Uganda: are reforms benefitting the poor?. Int J Equity Health.

[6] Burnham GM, Pariyo G, Galiwango E, Wabwire-Mangen F (2004). Discontinuation of cost sharing in Uganda.. Bull World Health Organ.

[7] Masiye F, Chitah BM, McIntyre D (2010). From targeted exemptions to user fee abolition in health care: experience from rural Zambia.. Soc Sci Med.

[8] Xu K, Evans DB, Kadama P, Nabyonga J, Ogwal PO, Nabukhonzo P (2006). Understanding the impact of eliminating user fees: utilization and catastrophic health expenditures in Uganda.. Soc Sci Med.

[9] Ridde V, Haddad S, Heinmüller R (2013). Improving equity by removing healthcare fees for children in Burkina Faso. J Epidemiol Community Health.. J Epidemiol Community Health.

[10] Nabyonga Orem JN, Mugisha F, Kirunga C, Macq J, Criel B (2011). Abolition of user fees: the Uganda paradox.. Health Policy Plan.

[11] Gordon-Strachan G, Bailey W, Henry-Lee A, Barnett J, Lalta S, Alleyne D (2010). The impact of user fees for preventive health care—Jamaica.. Soc Econ Stud.

[12] World Health Organization. World Health Assembly. Available: http://www.who.int/mediacentre/events/governance/wha/en/. Accessed: 14 March 2017.

[13] McPake B Brikci N Cometto G Schmidt A Araujo E.Removing user fees: learning from international experience to support the processHealth Policy Plan 2011 26 Suppl 2 ii104 117 10.1093/heapol/czr064 22027915

[14] International Labour Office. World Labour Report 2000: Income security and social protection in a changing world. 2000. Available: http://www.ilo.org/public/english/standards/relm/gb/docs/gb279/pdf/esp-7.pdf. Accessed: 13 January 2017.

[15] Institute TCPR. Fee or free a survey? A survey of the no-user fee policy in public hospitals in Jamaica. 2013. Available: http://www.capricaribbean.com/documents/no-user-fee-policy-public-hospitals-jamaica. Accessed: 11 November 2015.

[16] De La Haye W, Alexis S (2012). The impact of a no-user-fee policy on the quality of patient care/service delivery in Jamaica.. West Indian Med J.

[17] Bratt JH, Weaver MA, Foreit J, De Vargas T, Janowitz B (2002). The impact of price changes on demand for family planning and reproductive health services in Ecuador.. Health Policy Plan.

[18] Campbell A. The abolition of user fees in the Jamaican public health system: impact on access, care provided and the work of the professional nurse [thesis]. Wellington: Victoria University of Wellington; 2013.

[19] World Bank. Indicators. Available: http://data.worldbank.org/indicator. Accessed: 12 July 2016.

[20] Chao S. Jamaica’s effort in improving universal access within fiscal constraints. 2013. Available: http://documents.worldbank.org/curated/en/408381468044133381/Jamaicas-effort-in-improving-universal-access-within-fiscal-constraints/. Accessed: 12 July 2016.

[21] Coombs M. Universal Coverage in Jamaica. 2013. Available: http://www2.paho.org/hq/index.php?option=com_docman&task=doc_view&gid=19100&Itemid=270. Accessed: 4 May 2017.

[22] World Health Organization. Designing health financing systems to reduce catastrophic health expenditure. 2005. Available: http://www.who.int/health_financing/pb_2.pdf. Accessed:13 January 2017.

[23] Atun R, De Andrade LOM, Almeida G, Cotlear D, Dmytraczenko T, Frenz P (2015). Health-system reform and universal health coverage in Latin America.. Lancet.

[24] Cotlear D, Gómez-Dantés O, Knaul F, Atun R, Barreto IC, Cetrángolo O (2015). Overcoming social segregation in health care in Latin America.. Lancet.

[25] James C, Morris SS, Keith R, Taylor A (2005). Impact on child mortality of removing user fees: simulation model.. BMJ.

[26] Rutherford ME, Mulholland K, Hill PC (2010). How access to health care relates to under-five mortality in sub-Saharan Africa: systematic review.. Trop Med Int Health.

[27] Almeida G, Sarti FM (2013). Measuring evolution of income-related inequalities in health and health care utilization in selected Latin American and Caribbean countries.. Rev Panam Salud Publica.

[28] Evans D, Hausladen S, Kosec K, Reese N. Community-based conditional cash transfers in Tanzania: results from a randomized trial. 2014. Available: https://openknowledge.worldbank.org/handle/10986/17220. Accessed. 13 January 2017.

[29] Levesque J-F, Harris MF, Russell G (2013). Patient-centred access to health care: conceptualising access at the interface of health systems and populations.. Int J Equity Health.

[30] Planning Institute of Jamaica. Jamaica Survey of Living Conditions. Kingston: PIOJ & STATIN: 2013.

[31] Su TT, Kouyaté B, Flessa S (2006). Catastrophic household expenditure for health care in a low-income society: a study from Nouna District, Burkina Faso.. Bull World Health Organ.

[32] Jamaica Ministry of Health. Child and maternal health in Jamaica. 2011. Available: http://www.commonwealthhealth.org/americas/jamaica/child_and_maternal_health_in_jamaica/. Accessed: 27 March 2016.

[33] Rudan I, El Arifeen S, Bhutta ZA, Black RE, Brooks A, Chan KY (2011). Setting research priorities to reduce global mortality from childhood pneumonia by 2015.. PLoS Med.

[34] Mwabu G, Mwanzia J, Liambila W (1995). User charges in government health facilities in Kenya: effect on attendance and revenue.. Health Policy Plan.

[35] Meuwissen LE (2002). Problems of cost recovery implementation in district health care: a case study from Niger.. Health Policy Plan.

[36] Pradhan M, Prescott N (2002). Social risk management options for medical care in Indonesia.. Health Econ.

[37] Wagstaff A, Doorslaer EV (2003). Catastrophe and impoverishment in paying for health care: with applications to Vietnam 1993-1998.. Health Econ.

[38] Deininger K, Mpuga P (2005). Economic and welfare impact of the abolition of health user fees: evidence from Uganda.. J Afr Econ.

