# Supplementary material for: User–fee–removal improves equity of children’s health care utilization and reduces families’ financial burden: evidence from Jamaica
Source: J Glob Health. 2017 Jun 19;7(1):010502. doi: 10.7189/jogh.07.010502 (PMC5481893; doi:10.7189/jogh.07.010502)
Supplement: Online Supplementary Document [file jogh-07-010502-s001.pdf]

Online Supplementary Document

Li et al. User–fee–removal improves equity of children's healthcare utilization and reduces families' financial burden: evidence from Jamaica

J Glob Health 2017;7:010502

Table S1. ITS regression on the impact of user-fee-removal policy on healthcare utilization among children aged less-than 18-years old and children aged less than 5-years old, OLS regressions

	Under 18 years old			Under 5 years old		
	1) Overall	2) In poverty	3) Not in poverty	4) Overall	5) In poverty	6) Not in poverty
Trend	0.019*	-0.021	0.028*	0.033	0.003	0.034
	(0.010)	(0.026)	(0.014)	(0.022)	(0.047)	(0.022)
Post	0.158**	0.095	0.147**	0.325*	0.433	0.236
	(0.062)	(0.222)	(0.059)	(0.159)	(0.330)	(0.156)
Post*trend	-0.011	0.016	-0.016	-0.036	-0.032	-0.029
	(0.008)	(0.032)	(0.009)	(0.024)	(0.058)	(0.020)
Age	-0.010***	-0.005	-0.011***	-0.036***	-0.038**	-0.036***
	(0.001)	(0.010)	(0.002)	(0.006)	(0.012)	(0.006)
Male	-0.012	-0.023	-0.005	-0.01	-0.048	0.008
	(0.016)	(0.065)	(0.023)	(0.027)	(0.080)	(0.030)
Head of the household	0.057	-0.457**	0.430***			
	(0.248)	(0.172)	(0.050)			
Enrolled in private health insurance	0.104***	0.089	0.117**	0.023	-0.467***	0.074
	(0.032)	(0.127)	(0.042)	(0.038)	(0.134)	(0.055)
Enrolled in public health insurance	0.138	0.286***	0.109	0.174**	0.407***	0.099
	(0.081)	(0.057)	(0.097)	(0.074)	(0.035)	(0.111)

Wealth (The poorest wealth quintile is the reference group)						
Poorer	0.041			0.065		
	(0.033)			(0.065)		
Middle	0.102***			0.122**		
	(0.023)			(0.051)		
Richer	0.143***			0.159**		
	(0.038)			(0.063)		
Richest	0.122**			0.172**		
	(0.041)			(0.068)		
Household size, members only	-0.007	-0.011	-0.012	-0.006	-0.007	-0.012
	(0.004)	(0.014)	(0.007)	(0.009)	(0.021)	(0.010)
Place of residence ("Rural" is the reference group)						
Urban	0.037	0.003	0.054	0.023	0.069	0.027
	(0.046)	(0.099)	(0.051)	(0.048)	(0.116)	(0.049)
Town	0.012	-0.019	0.028	0.007	-0.097	0.051
	(0.055)	(0.072)	(0.051)	(0.062)	(0.132)	(0.056)
Education level of the head of the household ("No education" is the reference group)						
Primary education (Grade 1-6)	-0.105***	-0.295***	-0.001	-0.126***	-0.363***	0.031
	(0.021)	(0.080)	(0.046)	(0.036)	(0.099)	(0.048)
Secondary education (Grade 7-13)	-0.086**	-0.220***	-0.012	-0.056	-0.191**	0.02
	(0.039)	(0.040)	(0.064)	(0.040)	(0.065)	(0.060)
Higher education (Grade 13+)	-0.119**	-0.165*	-0.059	-0.082	-0.161**	-0.008
	(0.048)	(0.084)	(0.065)	(0.052)	(0.058)	(0.067)
cons	0.494***	0.868***	0.482***	0.461**	0.834**	0.495**
	(0.066)	(0.178)	(0.078)	(0.168)	(0.286)	(0.162)
N	1931	441	1488	959	237	722

Note:

1. We excluded all observations interviewed within four weeks after the interview date to make sure there is no confusion over whether the illnesses happened before or after the policy took place.
2. The design of JSLC is a two-stage stratified random sampling design, with the first stage a selection of Primary Sampling Units (PSUs), and the second stage a selection of dwellings. Standard errors are clustered at sampling region level, which is one level above the PSUs. Two PSUs were grouped into one sampling region. The robust standard

errors are reported in parentheses.

3. The education level of the household is obtained through the following approach: If the education level of the household head is available, we use it directly; if not available, we use the education level of the spouse of the household head instead; if still not available, we use the maximum education level of the household member instead; if still not available, we use the maximum education of the dwelling instead.
4. ***, **, * represents significance at the 1%, 5%, and 10% level respectively.
5. SEs are presented in the parenthesis.

Table S2. ITS regression on the impact of user-fee-removal policy on healthcare utilization among children aged less than 18-years old, year 1996-2012

	OLS			Logit		
	1)	2)	3)	4)	5)	6)
	Overall	In poverty	Not in poverty	Overall	In poverty	Not in poverty
Trend	-0.008	-0.018	0.012	-0.033	-0.075	0.051
	(0.010)	(0.028)	(0.010)	(0.061)	(0.117)	(0.044)
Level change after user-fee-removal policy (Post)	0.079*	0.058	0.158***	0.358	0.242	0.692***
	(0.048)	(0.107)	(0.037)	(0.373)	(0.445)	(0.161)
Trend change after user-fee-removal policy (Post*trend)	0.007	0.018	-0.014	0.027	0.072	-0.058
	(0.010)	(0.028)	(0.010)	(0.062)	(0.116)	(0.043)
Age	-0.011***	-0.007	-0.010***	-0.045***	-0.029	-0.042***
	(0.002)	(0.006)	(0.001)	(0.005)	(0.024)	(0.007)
Male	0.009	0.03	0.005	0.039	0.123	0.022
	(0.015)	(0.047)	(0.010)	(0.050)	(0.190)	(0.044)
Head of the household	0.21	-0.333***	0.462***	0.929*	0	0
	(0.248)	(0.059)	(0.065)	(0.478)	(.)	(.)
Enrolled in private health insurance	0.077***	0.14	0.078**	0.371**	0.614	0.360**
	(0.027)	(0.094)	(0.030)	(0.155)	(0.433)	(0.151)
Enrolled in public health insurance	-0.021	-0.02	-0.019	-0.084	-0.083	-0.069
	(0.023)	(0.047)	(0.027)	(0.086)	(0.191)	(0.115)

Wealth (The poorest wealth quintile is the reference group)						
Poorer	-0.01			-0.04		
	(0.024)			(0.116)		
Middle	0.071***			0.295***		
	(0.024)			(0.070)		
Richer	0.122***			0.522***		
	(0.026)			(0.187)		
Richest	0.120***			0.516***		
	(0.029)			(0.132)		
Household size, members only	0.001	0.004	-0.009***	0.004	0.015	-0.040***
	(0.003)	(0.004)	(0.003)	(0.012)	(0.017)	(0.012)
Place of residence ("Rural" is the reference group)						
Urban	0.025	0.032	0.043	0.109	0.135	0.187
	(0.019)	(0.087)	(0.030)	(0.149)	(0.361)	(0.133)
Town	-0.01	0.007	0.015	-0.042	0.027	0.063
	(0.022)	(0.053)	(0.033)	(0.161)	(0.217)	(0.137)
Education level of the head of the household ("No education" is the reference group)						
Primary education (Grade 1-6)	-0.029	-0.112**	-0.034	-0.116	-0.457**	-0.137
	(0.029)	(0.051)	(0.035)	(0.119)	(0.214)	(0.151)
Secondary education (Grade 7-13)	-0.034	-0.075	-0.044	-0.134	-0.31	-0.176
	(0.029)	(0.053)	(0.035)	(0.144)	(0.222)	(0.151)
Higher education (Grade 13+)	-0.042	0.004	-0.036	-0.167	0.022	-0.141
	(0.033)	(0.107)	(0.033)	(0.115)	(0.447)	(0.141)
cons	0.623***	0.595**	0.662***	0.491	0.384	0.662***
	(0.064)	(0.208)	(0.030)	(0.380)	(0.863)	(0.128)
r2	0.043	0.032	0.037			
N	3920	881	3545	3920	880	3543

Note:

1. We excluded all observations interviewed within four weeks after the interview date to make sure there is no confusion over whether the illnesses happened before or after the policy took place.
2. The design of JSLC is a two-stage stratified random sampling design, with the first stage a selection of Primary Sampling Units (PSUs), and the second stage a selection of dwellings. Standard errors are clustered at sampling region level, which is one level above the PSUs. Two PSUs were grouped into one sampling region. The robust standard

errors are reported in parentheses.

3. The education level of the household is obtained through the following approach: If the education level of the household head is available, we use it directly; if not available, we use the education level of the spouse of the household head instead; if still not available, we use the maximum education level of the household member instead; if still not available, we use the maximum education of the dwelling instead.
4. ***, **, * represents significance at the 1%, 5%, and 10% level respectively.
5. SEs are presented in the parenthesis.

Table S3. ITS regression on the impact of user-fee-removal policy on healthcare utilization among children aged less than 5-years old, year 1996-2012

	OLS			Logit		
	1)	2)	3)	4)	5)	6)
	Overall	In poverty	Not in poverty	Overall	In poverty	Not in poverty
Trend	0.022	0.021	0.016	0.112	0.099	0.081
	(0.023)	(0.031)	(0.014)	(0.111)	(0.137)	(0.063)
Level change after user-fee-removal policy (Post)	0.256	0.219	0.226*	1.246	0.966	1.118*
	(0.171)	(0.262)	(0.113)	(0.866)	(1.130)	(0.608)
Trend change after user-fee-removal policy (Post*trend)	-0.026	-0.023	-0.02	-0.131	-0.105	-0.103
	(0.026)	(0.038)	(0.017)	(0.130)	(0.164)	(0.084)
Age	-0.041***	-0.053**	-0.031***	-0.189***	-0.230***	-0.145***
	(0.009)	(0.018)	(0.007)	(0.044)	(0.083)	(0.035)
Male	0.012	-0.063	0.023	0.058	-0.28	0.107
	(0.023)	(0.056)	(0.024)	(0.109)	(0.232)	(0.110)
Enrolled in private health insurance	0.072	-0.071	0.106**	0.391*	-0.307	0.552**
	(0.044)	(0.060)	(0.047)	(0.236)	(0.251)	(0.264)

Enrolled in public health insurance	0.097**	0.075	0.056	0.486**	0.337	0.295
	(0.043)	(0.129)	(0.049)	(0.227)	(0.550)	(0.247)
Wealth (The poorest wealth quintile is the reference group)						
Poorer	0.037			0.161		
	(0.040)			(0.172)		
Middle	0.106**			0.474***		
	(0.037)			(0.164)		
Richer	0.165***			0.767***		
	(0.053)			(0.245)		
Richest	0.140**			0.657**		
	(0.061)			(0.324)		
Household size, members only	0.003	-0.004	-0.003	0.013	-0.018	-0.013
	(0.005)	(0.012)	(0.006)	(0.023)	(0.054)	(0.025)
Place of residence ("Rural" is the reference group)						
Urban	0.056	0.028	0.071*	0.273	0.112	0.342*
	(0.042)	(0.096)	(0.037)	(0.204)	(0.415)	(0.190)
Town	-0.008	-0.091	0.041	-0.039	-0.395	0.184
	(0.047)	(0.089)	(0.043)	(0.219)	(0.389)	(0.198)
Education level of the head of the household ("No education" is the reference group)						
Primary education (Grade 1-6)	-0.168***	-0.340***	-0.090**	-0.770***	-1.512***	-0.396**
	(0.040)	(0.089)	(0.040)	(0.191)	(0.426)	(0.173)
Secondary education (Grade 7-13)	-0.079	-0.189***	-0.050**	-0.375	-0.852***	-0.226***
	(0.045)	(0.053)	(0.020)	(0.229)	(0.246)	(0.088)
Higher education (Grade 13+)	-0.091*	-0.141**	-0.043	-0.435*	-0.642**	-0.201
	(0.045)	(0.063)	(0.049)	(0.229)	(0.279)	(0.227)
cons	0.541***	0.751***	0.621***	0.109	1.079	0.458
	(0.140)	(0.190)	(0.067)	(0.672)	(0.833)	(0.323)
r2	0.061	0.082	0.041			
N	1901	424	1767	1901	424	1767

Note:

1. We excluded all observations interviewed within four weeks after the interview date to make sure there is no confusion over whether the illnesses happened before or after the policy took place.
2. The design of JSLC is a two-stage stratified random sampling design, with the first stage a selection of Primary Sampling Units (PSUs), and the second stage a selection of dwellings. Standard errors are clustered at sampling region level, which is one level above the PSUs. Two PSUs were grouped into one sampling region. The robust standard errors are reported in parentheses.
3. The education level of the household is obtained through the following approach: If the education level of the household head is available, we use it directly; if not available, we use the education level of the spouse of the household head instead; if still not available, we use the maximum education level of the household member instead; if still not available, we use the maximum education of the dwelling instead.
4. ***, **, * represents significance at the 1%, 5%, and 10% level respectively.
5. SEs are presented in the parenthesis.

Table S4. ITS regression on the impact of user-fee-removal policy on out-of-pocket healthcare expenditure as a share of the household's non-food consumption, year 1996-2012

	Under 18 years old			Under 5 years old		
	1)	2)	3)	4)	5)	6)
	Overall	In poverty	Not in poverty	Overall	In poverty	Not in poverty
Trend	-0.002	-0.006	0.001	-0.001	-0.003	0.002
	(0.003)	(0.005)	(0.002)	(0.004)	(0.005)	(0.002)
Level change after user-fee-removal policy (Post)	-0.056***	-0.118***	-0.031	-0.070***	-0.116***	-0.043**
	(0.014)	(0.035)	(0.020)	(0.018)	(0.031)	(0.015)
Trend change after user-fee-removal policy (Post*trend)	0.004	0.014**	0.000	0.005	0.012**	0.000
	(0.003)	(0.005)	(0.003)	(0.004)	(0.005)	(0.003)
Age	-0.001***	-0.001	-0.001***	-0.002	-0.006***	-0.001
	0.000	(0.001)	0.000	(0.002)	(0.002)	(0.001)
Male	-0.001	0.002	0.000	-0.002	0.004	-0.001
	(0.003)	(0.009)	(0.003)	(0.006)	(0.010)	(0.005)
Head of the household	0.044	-0.064**	0.103***			
	(0.033)	(0.026)	(0.006)			
Enrolled in private health insurance	-0.008	0.027	-0.020***	-0.011	-0.029**	-0.023**
	(0.005)	(0.028)	(0.005)	(0.010)	(0.010)	(0.008)

Enrolled in public health insurance	-0.008	-0.014	-0.005	-0.007	0.004	-0.005
	(0.005)	(0.016)	(0.004)	(0.008)	(0.014)	(0.008)
Wealth (The poorest wealth quintile is the reference group)						
Poorer	-0.004			-0.006		
	(0.006)			(0.008)		
Middle	0.001			0.004		
	(0.008)			(0.009)		
Richer	-0.003			-0.006		
	(0.009)			(0.013)		
Richest	-0.021**			-0.028**		
	(0.009)			(0.011)		
Household size, members only	-0.005***	-0.003	-0.006***	-0.006***	-0.003*	-0.007***
	(0.001)	(0.002)	(0.001)	(0.001)	(0.001)	(0.001)
Place of residence ("Rural" is the reference group)						
Urban	-0.002	-0.01	-0.005	0.002	-0.001	-0.002
	(0.003)	(0.016)	(0.003)	(0.003)	(0.024)	(0.005)
Town	-0.009***	-0.001	-0.007**	-0.005	0.000	-0.002
	(0.002)	(0.008)	(0.003)	(0.003)	(0.006)	(0.007)
Education level of the head of the household ("No education" is the reference group)						
Primary education (Grade 1-6)	-0.01	-0.021	-0.01	-0.017	-0.035*	-0.012
	(0.007)	(0.012)	(0.006)	(0.012)	(0.016)	(0.009)
Secondary education (Grade 7-13)	-0.014**	-0.022**	-0.012**	-0.014*	-0.023	-0.013
	(0.005)	(0.009)	(0.005)	(0.006)	(0.014)	(0.009)
Higher education (Grade 13+)	-0.012**	0.004	-0.014	-0.012*	-0.009	-0.013
	(0.005)	(0.011)	(0.008)	(0.007)	(0.013)	(0.012)
cons	0.132***	0.131**	0.115***	0.141***	0.127**	0.128***
	(0.012)	(0.046)	(0.007)	(0.020)	(0.043)	(0.015)
r2	0.051	0.066	0.052	0.07	0.076	0.071
N	3873	869	3500	1876	461	1699

Note:

1. We excluded all observations interviewed within four weeks after the interview date to make sure there is no confusion over whether the illnesses happened before or after the policy took place.

2. The design of JSLC is a two-stage stratified random sampling design, with the first stage a selection of Primary Sampling Units (PSUs), and the second stage a selection of dwellings. Standard errors are clustered at sampling region level, which is one level above the PSUs. Two PSUs were grouped into one sampling region. The robust standard errors are reported in parentheses.
3. The education level of the household is obtained through the following approach: If the education level of the household head is available, we use it directly; if not available, we use the education level of the spouse of the household head instead; if still not available, we use the maximum education level of the household member instead; if still not available, we use the maximum education of the dwelling instead.
4. We dropped 1% of individuals with the highest healthcare cost (outliers).
5. ***, **, * represents significance at the 1%, 5%, and 10% level respectively.
6. SEs are presented in the parenthesis.

Table S5. Falsification tests on the impact of user-fee-removal policy on healthcare utilization and the households' financial burden, assuming the user-fee-removal policy targeted at adults over 18, OLS regressions

	Y=whether the children visit a health practitioner if fell ill in the past 4 weeks			Y=the children's out-of-pocket healthcare expenditure as a share of household's non-food consumption		
	1) Overall	2) In poverty	3) Not in poverty	4) Overall	5) In poverty	6) Not in poverty
Trend	0.016	0.034	0.011	0	0.007	-0.001
	(0.010)	(0.034)	(0.011)	(0.002)	(0.012)	(0.002)
Level change after user-fee-removal policy (Post)	0.015	0.048	-0.013	-0.009	0.03	-0.02
	(0.049)	(0.185)	(0.069)	(0.014)	(0.047)	(0.016)
Trend change after user-fee-removal policy (Post*trend)	-0.002	-0.012	0.003	-0.002	-0.009	0
	(0.009)	(0.032)	(0.012)	(0.002)	(0.011)	(0.002)
Age	0.001	0	0.001	0.001***	0	0.001***
	(0.001)	(0.001)	(0.001)	(0.000)	(0.000)	(0.000)
Male	-0.051**	-0.024	-0.053**	-0.005*	0.001	-0.006
	(0.023)	(0.046)	(0.021)	(0.002)	(0.011)	(0.004)
Head of the household	0.008	0.008	0.007	0.005**	-0.011	0.007***
	(0.017)	(0.059)	(0.013)	(0.002)	(0.014)	(0.002)

Enrolled in private health insurance	0.112***	0.089	0.123***	-0.010**	0.022	-0.010**
	(0.019)	(0.064)	(0.023)	(0.003)	(0.014)	(0.003)
Enrolled in public health insurance	0.044	0.058	0.046	-0.01	-0.001	-0.011
	(0.029)	(0.077)	(0.032)	(0.006)	(0.019)	(0.008)
Wealth (The poorest wealth quintile is the reference group)						
Poorer	0.041**			0.015**		
	(0.014)			(0.006)		
Middle	0.075***			0.015**		
	(0.010)			(0.006)		
Richer	0.094***			0.018***		
	(0.014)			(0.004)		
Richest	0.124***			0.022***		
	(0.016)			(0.003)		
Household size, members only	0.015***	0.020**	0.007**	-0.003***	0.003**	-0.006***
	(0.003)	(0.006)	(0.003)	(0.001)	(0.001)	(0.001)
Place of residence ("Rural" is the reference group)						
Urban	-0.012	0.042	-0.007	-0.01	-0.007	-0.009
	(0.027)	(0.052)	(0.025)	(0.006)	(0.016)	(0.005)
Town	-0.008	0.091	-0.012	-0.011*	-0.008	-0.01
	(0.024)	(0.051)	(0.027)	(0.006)	(0.009)	(0.006)
Education level of the head of the household ("No education" is the reference group)						
Primary education (Grade 1-6)	0.041	0.056	0.043	0.007	-0.003	0.008
	(0.048)	(0.124)	(0.040)	(0.013)	(0.023)	(0.012)
Secondary education (Grade 7-13)	0.011	-0.01	0.022	0.013	0.016	0.011
	(0.046)	(0.128)	(0.047)	(0.013)	(0.021)	(0.012)
Higher education (Grade 13+)	-0.059	-0.041	-0.038	-0.001	0.009	0
	(0.059)	(0.076)	(0.054)	(0.013)	(0.033)	(0.013)
cons	0.336***	0.153	0.481***	0.055***	-0.016	0.092***
	(0.093)	(0.191)	(0.081)	(0.015)	(0.050)	(0.014)
r2	0.057	0.086	0.049	0.065	0.034	0.081
N	4165	595	3570	4165	595	3570

Note:

1. We excluded all observations interviewed within four weeks after the interview date to make sure there is no confusion over whether the illnesses happened before or after the policy took place.
2. The design of JSLC is a two-stage stratified random sampling design, with the first stage a selection of Primary Sampling Units (PSUs), and the second stage a selection of dwellings. Standard errors are clustered at sampling region level, which is one level above the PSUs. Two PSUs were grouped into one sampling region. The robust standard errors are reported in parentheses.
3. The education level of the household is obtained through the following approach: If the education level of the household head is available, we use it directly; if not available, we use the education level of the spouse of the household head instead; if still not available, we use the maximum education level of the household member instead; if still not available, we use the maximum education of the dwelling instead.
4. ***, **, * represents significance at the 1%, 5%, and 10% level respectively.
5. SEs are presented in the parenthesis.

Table S6. Falsification tests on the impact of user-fee-removal policy on healthcare utilization among children aged less than 18-years, assuming the user-fee-removal policy was implemented on May 28th, 2006

	OLS			Logit		
	1)	2)	3)	4)	5)	6)
	Overall	In poverty	Not in poverty	Overall	In poverty	Not in poverty
Trend	-0.006	-0.006	-0.006	-0.02	-0.021	-0.023
	(0.049)	(0.060)	(0.050)	(0.211)	(0.286)	(0.209)
Level change after user-fee-removal policy (Post)	-0.014	-0.067	-0.033	-0.06	-0.223	-0.166
	(0.197)	(0.269)	(0.215)	(0.861)	(1.295)	(0.934)
Trend change after user-fee-removal policy (Post*trend)	0.021	0.03	0.022	0.091	0.12	0.096
	(0.050)	(0.053)	(0.053)	(0.217)	(0.259)	(0.227)
Age	-0.009***	-0.004	-0.009***	-0.042***	-0.02	-0.042***
	(0.002)	(0.008)	(0.002)	(0.008)	(0.036)	(0.009)
Male	-0.021	-0.081	-0.002	-0.095	-0.359	-0.006
	(0.020)	(0.054)	(0.028)	(0.093)	(0.234)	(0.128)
Head of the household	-0.157	0	-0.196	-0.684	0	-0.82
	(0.233)	(.)	(0.250)	(1.146)	(.)	(1.206)
Enrolled in private health insurance	0.109***	0.148	0.120***	0.553***	0.624	0.605***

	(0.028)	(0.119)	(0.037)	(0.154)	(0.536)	(0.200)
Enrolled in public health insurance	0.138	-0.048	0.152	0.655	-0.247	0.737
	(0.090)	(0.342)	(0.086)	(0.487)	(1.375)	(0.483)
Wealth (The poorest wealth quintile is the reference group)						
Poorer	0.035			0.148		
	(0.037)			(0.157)		
Middle	0.082*			0.356**		
	(0.038)			(0.164)		
Richer	0.129**			0.579***		
	(0.047)			(0.217)		
Richest	0.099**			0.442**		
	(0.042)			(0.196)		
Household size, members only	-0.009**	-0.014	-0.012	-0.039**	-0.064	-0.053*
	(0.004)	(0.012)	(0.007)	(0.017)	(0.048)	(0.031)
Place of residence ("Rural" is the reference group)						
Urban	0.029	-0.012	0.048	0.131	-0.045	0.227
	(0.044)	(0.086)	(0.048)	(0.205)	(0.367)	(0.232)
Town	0.022	0.034	0.03	0.102	0.16	0.136
	(0.064)	(0.081)	(0.054)	(0.287)	(0.340)	(0.243)
Education level of the head of the household ("No education" is the reference group)						
Primary education (Grade 1-6)	-0.064	-0.319***	0.041	-0.297	-1.577***	0.197
	(0.037)	(0.072)	(0.055)	(0.183)	(0.203)	(0.244)
Secondary education (Grade 7-13)	-0.057	-0.279***	0.037	-0.268	-1.404***	0.18
	(0.036)	(0.037)	(0.049)	(0.174)	(0.290)	(0.221)
Higher education (Grade 13+)	-0.083**	-0.23	0.002	-0.392***	-1.198	0.015
	(0.031)	(0.131)	(0.066)	(0.151)	(0.735)	(0.304)
cons	0.559**	0.779**	0.560**	0.233	1.375	0.204
	(0.194)	(0.295)	(0.218)	(0.828)	(1.283)	(0.910)
r2	0.061	0.088	0.049			
N	1960	332	1628	1952	331	1621

Note:

1. We excluded all observations interviewed within four weeks after May 28th, 2006, to make sure there is no confusion over whether the illnesses happened before or after the policy took place.
2. The design of JSLC is a two-stage stratified random sampling design, with the first stage a selection of Primary Sampling Units (PSUs), and the second stage a selection of dwellings. Standard errors are clustered at sampling region level, which is one level above the PSUs. Two PSUs were grouped into one sampling region. The robust standard errors are reported in parentheses.
3. The education level of the household is obtained through the following approach: If the education level of the household head is available, we use it directly; if not available, we use the education level of the spouse of the household head instead; if still not available, we use the maximum education level of the household member instead; if still not available, we use the maximum education of the dwelling instead.
4. ***, **, * represents significance at the 1%, 5%, and 10% level respectively.
5. SEs are presented in the parenthesis.

Table S7. Falsification tests on the impact of user-fee-removal policy on healthcare utilization among children aged less than 5-years, assuming the user-fee-removal policy was implemented on May 28th, 2006

	OLS			Logit		
	1)	2)	3)	4)	5)	6)
	Overall	In poverty	Not in poverty	Overall	In poverty	Not in poverty
Trend	0.007	0.054	-0.015	0.036	0.287	-0.059
	(0.064)	(0.085)	(0.091)	(0.267)	(0.362)	(0.376)
Level change after user-fee-removal policy (Post)	0.061	0.016	-0.028	0.294	0.22	-0.114
	(0.212)	(0.381)	(0.325)	(0.879)	(1.638)	(1.338)
Trend change after user-fee-removal policy (Post*trend)	0.004	-0.03	0.026	0.014	-0.17	0.113
	(0.059)	(0.084)	(0.087)	(0.246)	(0.355)	(0.360)
Age	-0.040***	-0.096***	-0.026**	-0.188***	-0.439***	-0.126***
	(0.007)	(0.027)	(0.009)	(0.038)	(0.115)	(0.042)
Male	-0.011	-0.087	0.014	-0.046	-0.434	0.075
	(0.031)	(0.055)	(0.042)	(0.145)	(0.275)	(0.197)
Enrolled in private health insurance	0.049	-0.329**	0.082	0.248	-1.584***	0.424
	(0.040)	(0.120)	(0.051)	(0.203)	(0.563)	(0.271)
Enrolled in public health insurance	0.139	0.165	0.144	0.702	0.769	0.73
	(0.091)	(0.204)	(0.093)	(0.516)	(0.843)	(0.539)

Wealth (The poorest wealth quintile is the reference group)						
Poorer	0.11			0.469*		
	(0.063)			(0.276)		
Middle	0.148**			0.654**		
	(0.060)			(0.275)		
Richer	0.183**			0.835***		
	(0.063)			(0.302)		
Richest	0.183**			0.844**		
	(0.080)			(0.414)		
Household size, members only	-0.005	-0.013	-0.009	-0.022	-0.058	-0.041
	(0.007)	(0.018)	(0.007)	(0.031)	(0.082)	(0.034)
Place of residence ("Rural" is the reference group)						
Urban	0.029	0.051	0.046	0.132	0.26	0.223
	(0.047)	(0.085)	(0.047)	(0.220)	(0.361)	(0.231)
Town	0.056	-0.032	0.078	0.271	-0.155	0.383
	(0.080)	(0.110)	(0.071)	(0.372)	(0.509)	(0.343)
Education level of the head of the household ("No education" is the reference group)						
Primary education (Grade 1-6)	-0.016	-0.281**	0.082	-0.063	-1.390**	0.393
	(0.069)	(0.119)	(0.054)	(0.311)	(0.568)	(0.241)
Secondary education (Grade 7-13)	0.01	-0.215**	0.087	0.055	-1.055**	0.417*
	(0.061)	(0.083)	(0.049)	(0.287)	(0.432)	(0.219)
Higher education (Grade 13+)	0.004	-0.133	0.074	0.023	-0.69	0.352
	(0.068)	(0.203)	(0.060)	(0.319)	(0.965)	(0.276)
cons	0.444	0.859*	0.548	-0.299	1.562	0.117
	(0.278)	(0.438)	(0.393)	(1.152)	(1.845)	(1.637)
r2	0.072	0.173	0.047			
N	960	169	791	959	169	790

Note:

1. We excluded all observations interviewed within four weeks after May 28th, 2006, to make sure there is no confusion over whether the illnesses happened before or after the policy took place.
2. The design of JSLC is a two-stage stratified random sampling design, with the first stage a selection of Primary Sampling Units (PSUs), and the second stage a selection of dwellings. Standard errors are clustered at sampling region level, which is one level above the PSUs. Two PSUs were grouped into one sampling region. The robust standard

errors are reported in parentheses.

3. The education level of the household is obtained through the following approach: If the education level of the household head is available, we use it directly; if not available, we use the education level of the spouse of the household head instead; if still not available, we use the maximum education level of the household member instead; if still not available, we use the maximum education of the dwelling instead.
4. ***, **, * represents significance at the 1%, 5%, and 10% level respectively.
5. SEs are presented in the parenthesis.

Table S8. Falsification tests on the impact of user-fee-removal policy on out-of-pocket healthcare expenditure as a share of the household's non-food consumption, assuming the user-fee-removal policy was implemented on May 28th, 2006

	Under 18			Under 5		
	1)	2)	3)	4)	5)	6)
	Overall	In poverty	Not in poverty	Overall	In poverty	Not in poverty
Trend	-0.001	0.003	-0.005	0.009	0.035	-0.001
	(0.010)	(0.014)	(0.010)	(0.010)	(0.022)	(0.015)
Level change after user-fee-removal policy (Post)	-0.028	-0.042	-0.039	0.004	0.071	-0.027
	(0.048)	(0.081)	(0.046)	(0.040)	(0.120)	(0.058)
Trend change after user-fee-removal policy (Post*trend)	0.001	0.001	0.004	-0.009	-0.029	0
	(0.011)	(0.016)	(0.010)	(0.010)	(0.023)	(0.015)
Age	-0.001**	-0.001	-0.001**	-0.003*	-0.002	-0.003**
	0.000	(0.001)	0.000	(0.002)	(0.005)	(0.001)
Male	-0.004	-0.017	-0.002	-0.007	-0.023	-0.004
	(0.004)	(0.012)	(0.005)	(0.008)	(0.018)	(0.010)
Head of the household	0.012	0	0.003	0	0	0
	(0.064)	(.)	(0.063)	(.)	(.)	(.)
Enrolled in private health insurance	-0.01	0.042	-0.018**	-0.014	-0.046***	-0.016
	(0.007)	(0.024)	(0.008)	(0.011)	(0.010)	(0.011)

Enrolled in public health insurance	-0.01	-0.055**	-0.009	-0.008	-0.044**	-0.007
	(0.007)	(0.019)	(0.007)	(0.013)	(0.017)	(0.013)
Wealth (The poorest wealth quintile is the reference group)						
Poorer	-0.002			0		
	(0.006)			(0.005)		
Middle	0.007			0.01		
	(0.004)			(0.008)		
Richer	0.001			0.002		
	(0.011)			(0.012)		
Richest	-0.012			-0.01		
	(0.007)			(0.006)		
Household size, members only	-0.006***	-0.002	-0.007***	-0.005***	0	-0.007***
	(0.001)	(0.002)	(0.001)	(0.001)	(0.002)	(0.002)
Place of residence ("Rural" is the reference group)						
Urban	-0.008*	-0.013	-0.008*	-0.006	0	-0.010**
	(0.004)	(0.012)	(0.004)	(0.005)	(0.020)	(0.004)
Town	-0.006***	-0.007	-0.007***	0.002	-0.009	0.001
	(0.002)	(0.007)	(0.002)	(0.003)	(0.008)	(0.003)
Education level of the head of the household ("No education" is the reference group)						
Primary education (Grade 1-6)	-0.004	-0.013	0	-0.004	-0.041**	0.006
	(0.009)	(0.018)	(0.011)	(0.015)	(0.017)	(0.013)
Secondary education (Grade 7-13)	-0.009	-0.022*	-0.005	-0.007	-0.024**	-0.001
	(0.006)	(0.012)	(0.009)	(0.007)	(0.010)	(0.009)
Higher education (Grade 13+)	-0.007	0.007	-0.008	-0.006	-0.017	-0.001
	(0.006)	(0.007)	(0.009)	(0.008)	(0.010)	(0.010)
cons	0.119**	0.085	0.138***	0.08	-0.042	0.127*
	(0.047)	(0.071)	(0.044)	(0.049)	(0.133)	(0.066)
r2	0.069	0.084	0.079	0.076	0.093	0.093
N	1938	343	1595	1931	342	1589

Note:

1. We excluded all observations interviewed within four weeks after May 28th, 2006, to make sure there is no confusion over whether the illnesses happened before or after the policy took place.

2. The design of JSLC is a two-stage stratified random sampling design, with the first stage a selection of Primary Sampling Units (PSUs), and the second stage a selection of dwellings. Standard errors are clustered at sampling region level, which is one level above the PSUs. Two PSUs were grouped into one sampling region. The robust standard errors are reported in parentheses. The education level of the household is obtained through the following approach: If the education level of the household head is available, we use it directly; if not available, we use the education level of the spouse of the household head instead; if still not available, we use the maximum education level of the household member instead; if still not available, we use the maximum education of the dwelling instead.
3. ***, **, * represents significance at the 1%, 5%, and 10% level respectively.
4. SEs are presented in the parenthesis.
